# Supplementary material for: Periodic Mesoporous Organosilica Nanoparticles for CO2 Adsorption at Standard Temperature and Pressure
Source: Molecules. 2022 Jun 30;27(13):4245. doi: 10.3390/molecules27134245 (PMC9268279; doi:10.3390/molecules27134245)
Supplement: Supplementary file 1 [file molecules-27-04245-s001.zip › molecules-1772508-supplementary.pdf]

ESI: Periodic mesoporous organosilica nanoparticles for CO_2 adsorption at standard temperature and pressure

Paul Kirren [1], Lucile Barka [1], Saher Rahmani [1], Nicolas Bondon [1], Nicolas Donzel [2], Philippe Trens [2], Aurélie Bessière [2], Laurence Raehm [2], Clarence Charnay [2], and Jean-Olivier Durand [2],
[1] Capgemini Engineering ; paul.kirren@altran.com (P.K.); lucile.barka@altran.com (L.B.); saher.rahmani@altran.com (S.R.)
[2] ICGM, Univ Montpellier, CNRS, ENSCM, Montpellier, France; nicolas.bondon@umontpellier.fr (N.B.); nicolas.donzel@umontpellier.fr (N.D.): Philippe.Trens@enscm.fr (P.T.); aurelie.bessière@umontpellier.fr (A.B.); laurence.raehm@umontpellier.fr (L.R.); clarence.charnay@umontpellier.fr (C.C.); jean-olivier.durand@umontpellier.fr (J.-O.D.).

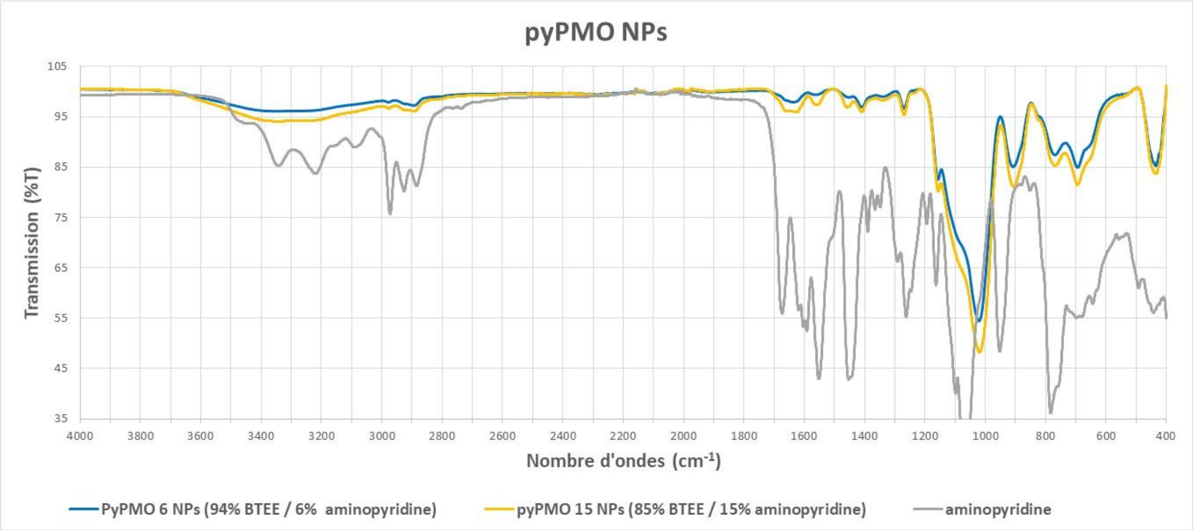

Figure S1. FTIR-ATR of pyPMO NPs

Table S1, Microanalyses of PMO1NPs and pyPMO NPs

PMO NPs	% C	% H	% N	% O	% Si
PMO 1 NPs (100% ethane)	18.298	4.358	0.156	6.099	71.089
pyPMO 6 NPs (94% ethane / 6% pyridine)	18.290	4.411	1.446	6.583	69.27
pyPMO 15 NPs (85% ethane / 15% pyridine)	19.386	4.279	2.880	5.987	67.468

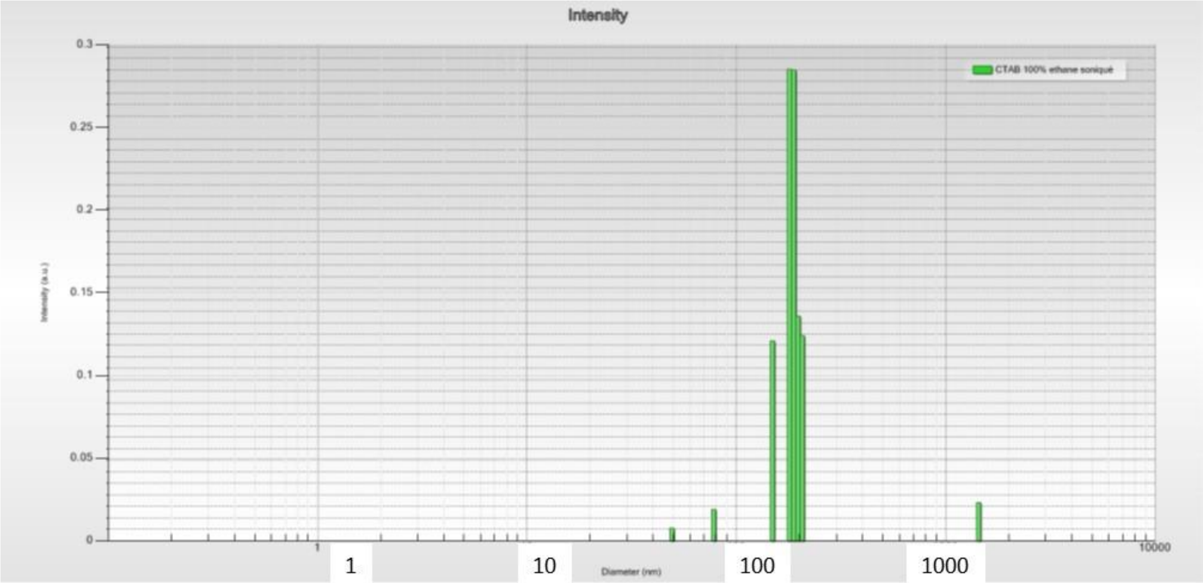

***Figure S2.** DLS of PMO 1 NPs in EtOH intensity mode

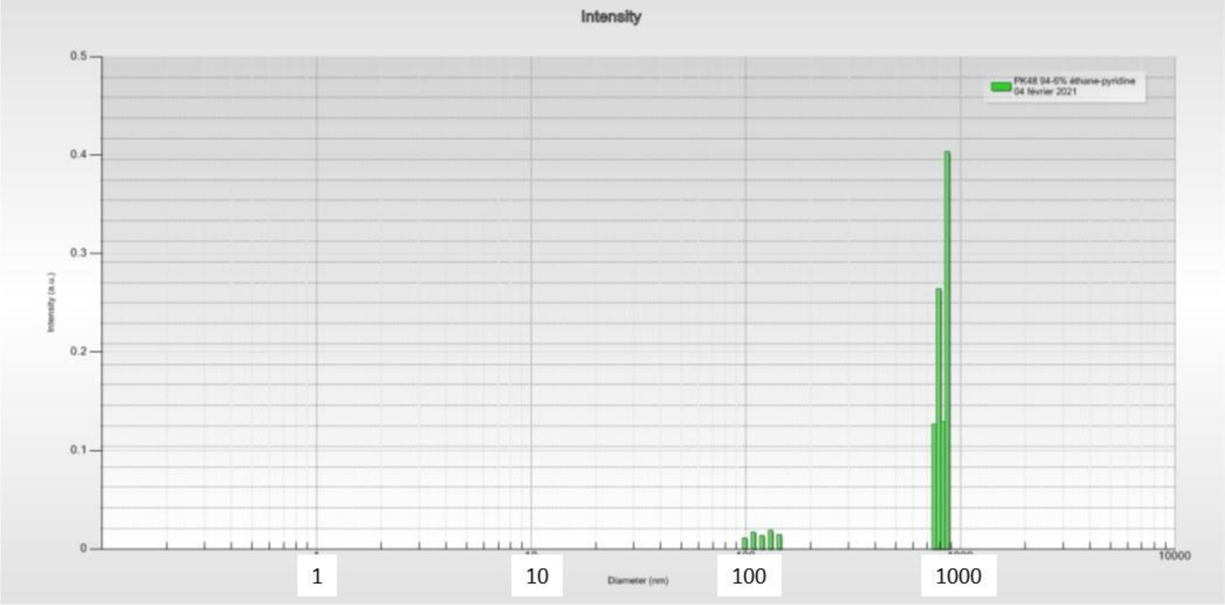

Figure S3. DLS of pyPMO 6 NPs in EtOH intensity mode

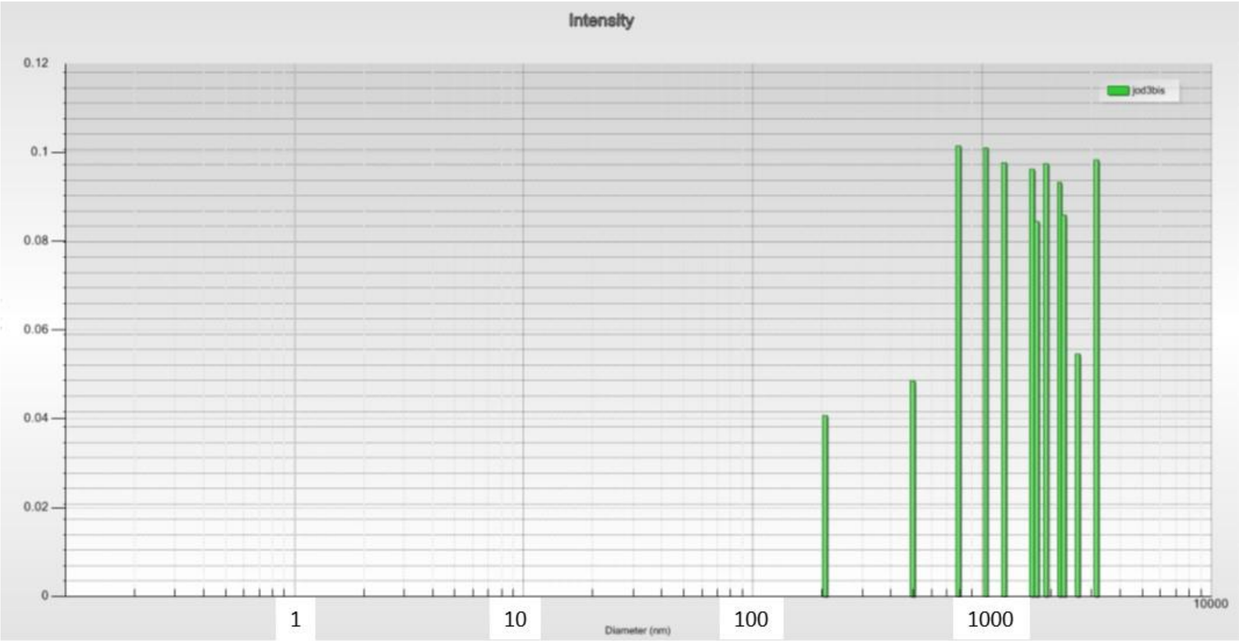

Figure S4. DLS of pyPMO 15 NPs in EtOH intensity mode

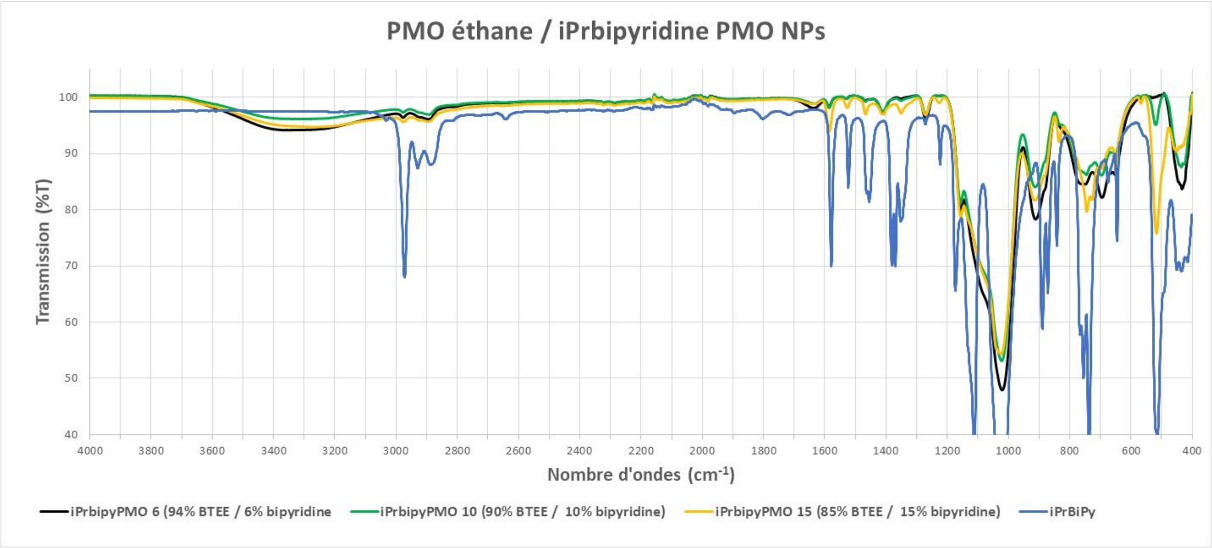

Figure S5. FTIR-ATR of iPrbipyPMO NPs

Table S2, Microanalyses of PMO1 NPs and iPrbipyPMO NPs

PMO NPs	% C	% H	% N	% O	% Si
PMO 1 NPs (100% ethane)	18.298	4.358	0.156	6.099	71.089
iPrbipyPMO 6 NPs (94/6% ethane/bipyridine)	19.374	3.934	0.976	3.143	72.573
iPrbipyPMO 10 NPs (90/10% ethane/bipyridine)	21.546	3.854	2.214	3.428	68.958

iPrbipyPMO 15 NPs (85/15% ethane/bipyridine)	28.011	3.923	4.315	4.466	59.285

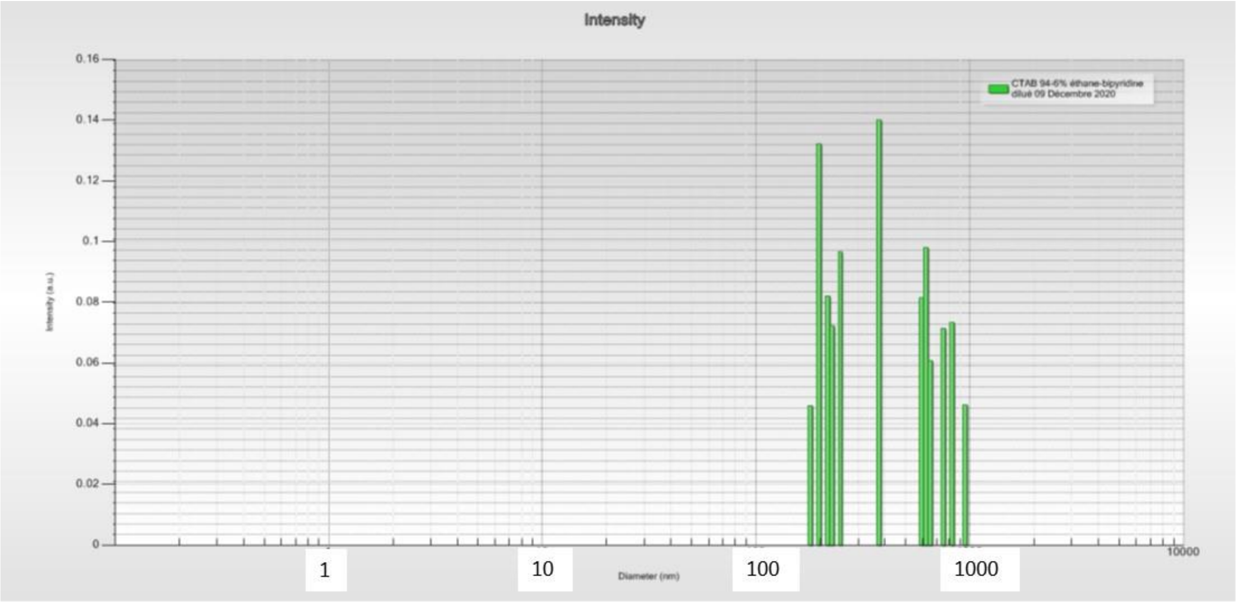

Figure S6. DLS of iPrbipyPMO 6 NPs in EtOH intensity mode

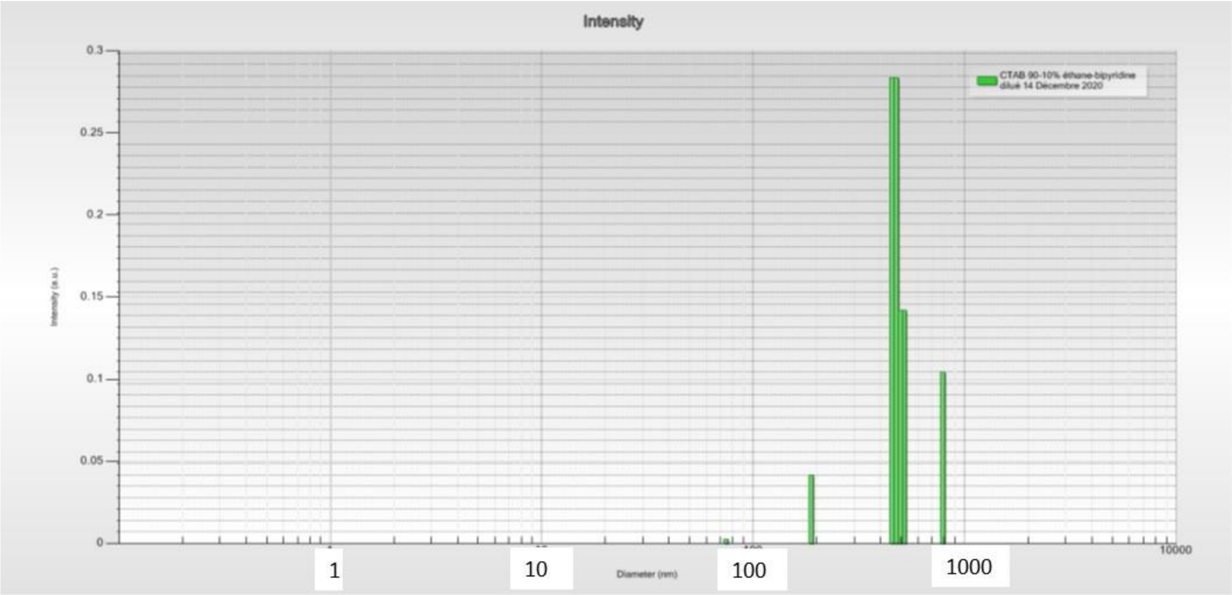

Figure S7. DLS of iPrbipyPMO 10 NPs in EtOH intensity mode

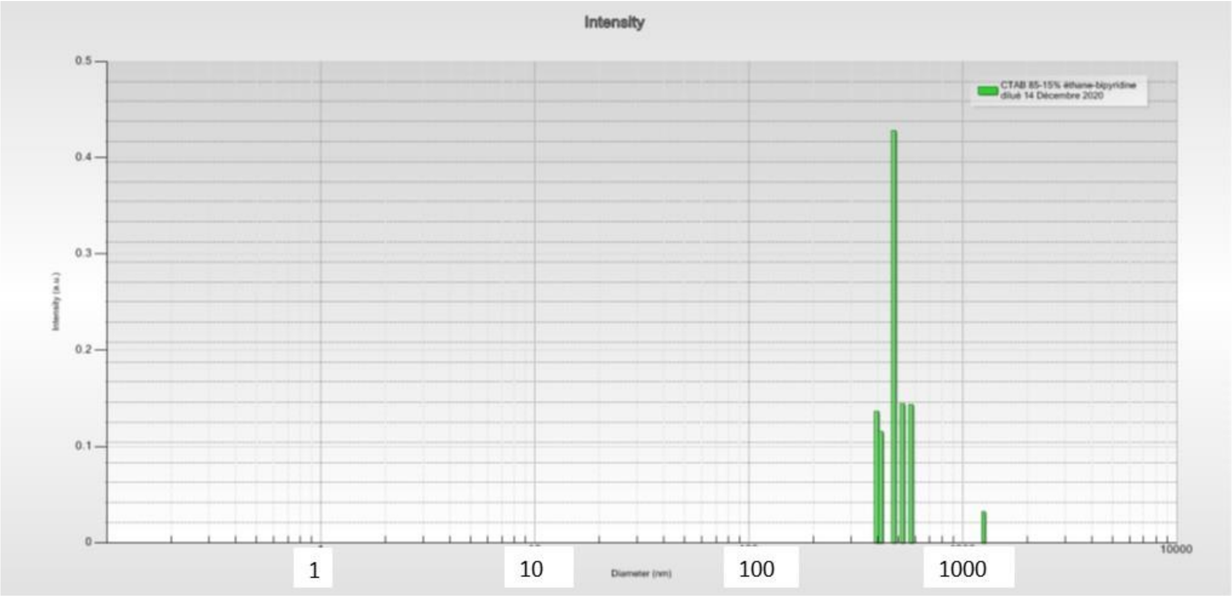

Figure S8. DLS of iPrbipyPMO 15 NPs in EtOH intensity mode

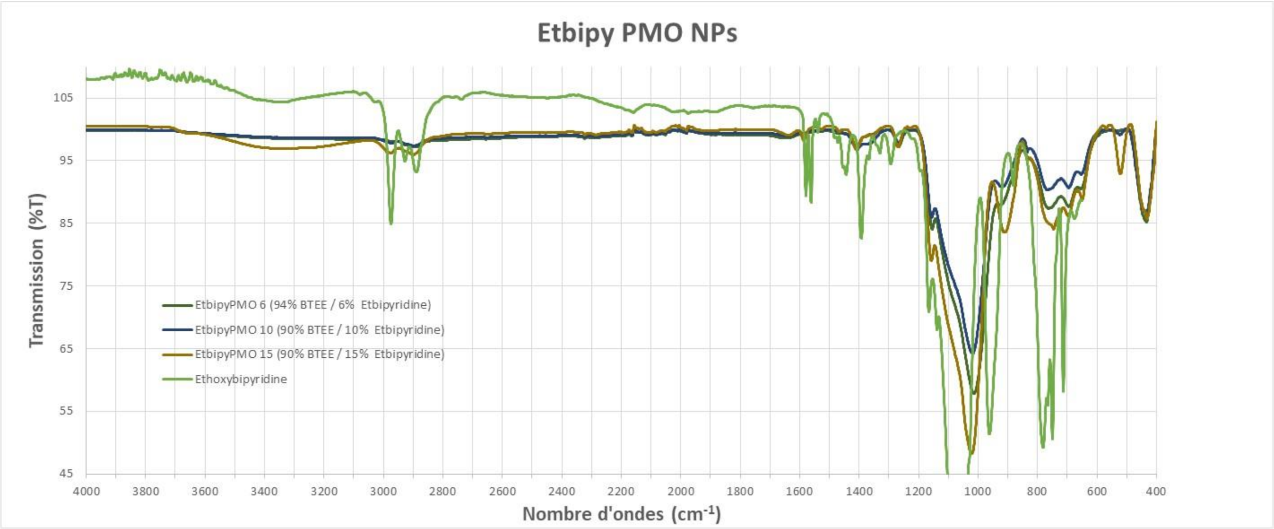

Figure S9. FTIR-ATR of EtbipyPMO NPs

Table S3, Microanalyses of PMO1 NPs and EtbipyPMO NPs

PMO NPs	% C	% N
PMO 1 NPs (100% éthane)	18.298	0.156
EtbipyPMO 6 NPs (94% éthane / 6% Etbipyridine)	14.710	0.461
EtbipyPMO 10 NPs (90% éthane / 10% Etbipyridine)	16.742	0.855
EtbipyPMO 15 NPs (85% éthane / 15% Etbipyridine)	18.162	1.670

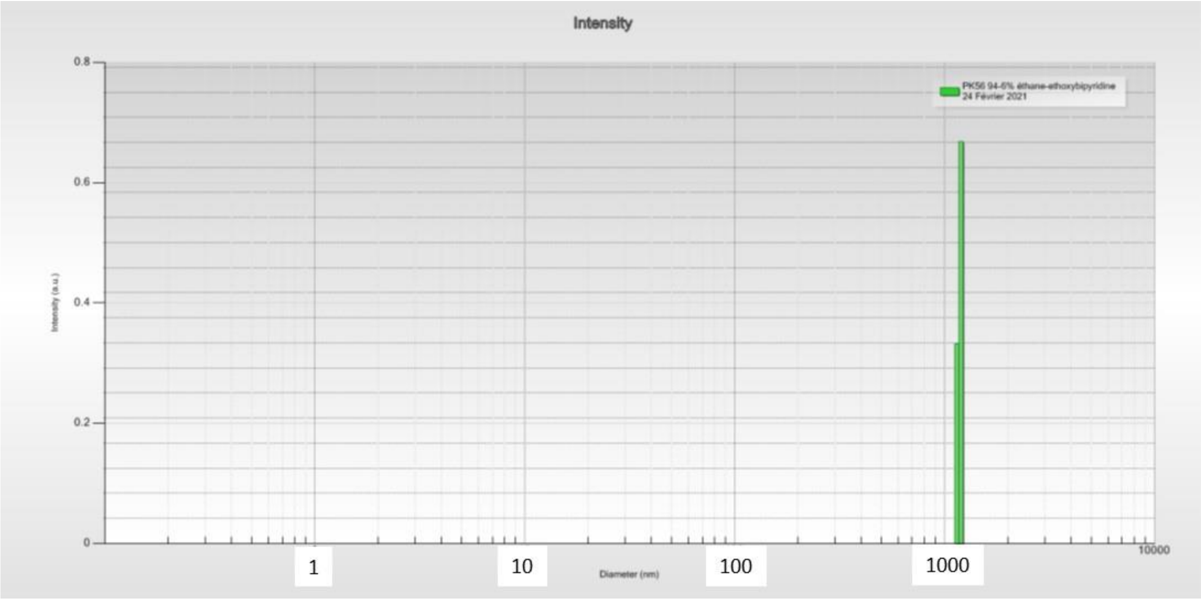

Figure S10. DLS of EtPrbipyPMO 6 NPs in EtOH intensity mode

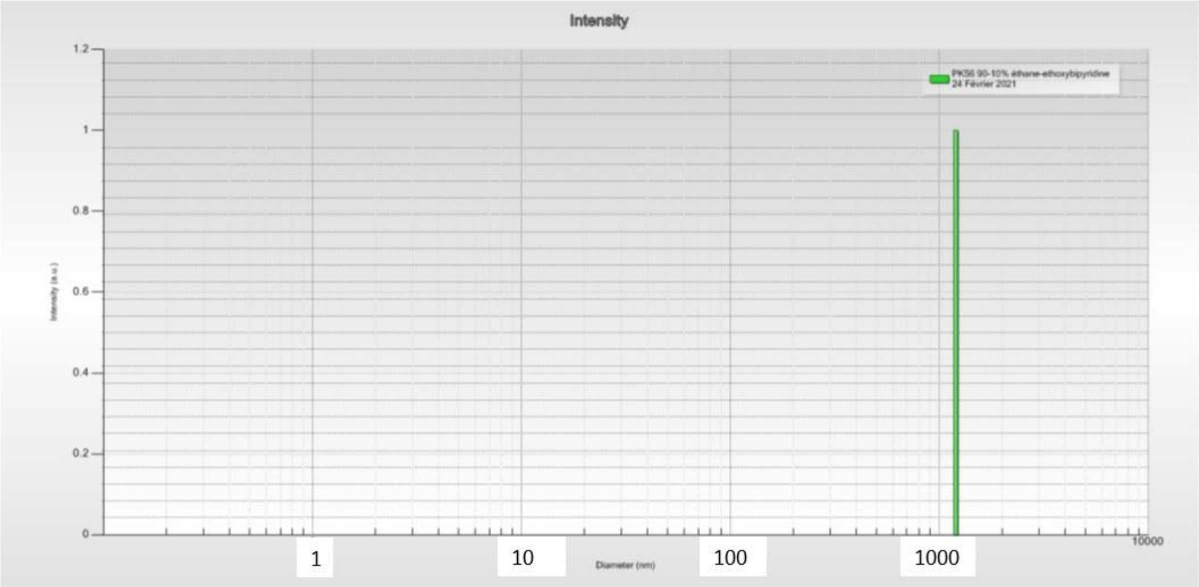

Figure S11. DLS of EtbipyPMO 10 NPs in EtOH intensity mode

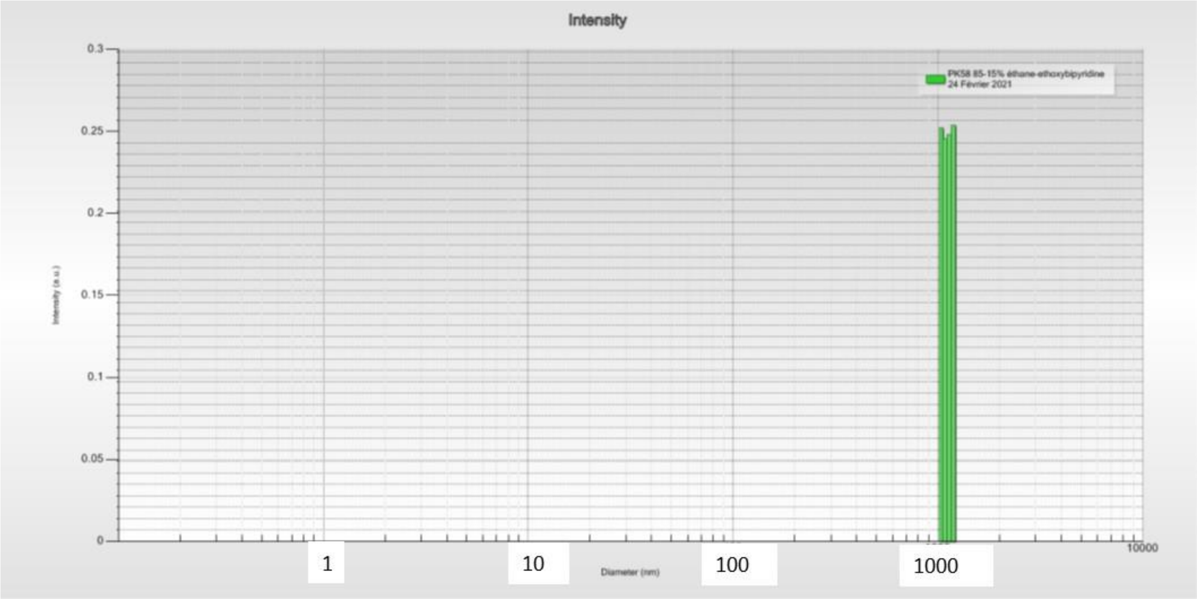

Figure S12. DLS of EtbipyPMO 15 NPs in EtOH intensity mode
